# Characteristics of Lightweight Cellular Concrete and Effects on Mechanical Properties

**DOI:** 10.3390/ma13122678

**Published:** 2020-06-12

**Authors:** Wei Yu, Xu Liang, Frank Mi-Way Ni, Abimbola Grace Oyeyi, Susan Tighe

**Affiliations:** 1 College of Traffic and Transportation, Chongqing Jiaotong University, Chongqing 400074, China; 2 Department of Civil & Environmental Engineering, University of Waterloo, Waterloo, ON N2L 3G1, Canada; a2olaleye@uwaterloo.ca (A.G.O.); sltighe@uwaterloo.ca (S.T.); 3 College of Architecture and Civil Engineering, Shangqiu Normal University, Shangqiu 476000, China; jesselx1230@gmail.com

**Keywords:** lightweight cellular concrete (LCC), pore structure, image processing, shape descriptors, mechanical properties

## Abstract

This study investigated the pore structure and its effects on mechanical properties of lightweight cellular concrete (LCC) in order to understand more and detailed characteristics of such structure. As part of investigation, environment scanning electron microscopes (ESEM) and industrial high-definition (HD) macro photography camera were separately used to capture and compare images of specimens. Physical properties of the pore structure, including pore area, size, perimeter, fit ellipse, and shape descriptors, were studied based on the image processing technology and software applications. Specimens with three different densities (400, 475, and 600 kg/m^3^) were prepared in the laboratory. Firstly, the effects of density on the characteristics of pore structure were investigated; furthermore, mechanical properties (compressive strength, modulus of elasticity and Poisson’s ratio, flexural strength and splitting tensile strength of LCC) were tested. The relationships among pore characteristics, density, and mechanical properties were analyzed. Based on the results obtained from the lab test—comparisons made between specimens with high-densities and those with low-densities—it was found significant variability in bubble size, thickness, and irregularity of pores. Furthermore, the increase of density is accompanied by better mechanical properties, and the main influencing factors are the thickness of the solid part and the shape of the bubble. The thicker of solid part and more regular pores of LCC has, the better mechanical properties are.

## 1. Introduction

Lightweight cellular concrete (LCC) is a porous material with a typical density ranging from 300 to 1800 kg/m^3^ [1,2,3,4,5,6], which contains a homogeneous air bubble structure in the mix. Other academic terms describing this material are foam concrete [7], low-density foam concrete, or cellular lightweight concrete, etc. [8,9]. It was first patented in 1923 [10] as a void filler material. The textural surface and microstructural cells make it widely used in the fields thermal insulation [11], sound absorbance [12], and fire resistance [13]. It is also used for bridge abutment filling [14], building foundations [15,16], and airport buffer systems [17]. Over the past 30 years, LCC has been widely used for bulk filling [4], ditch repair, retaining walls [18], bridge abutment backfill [19], slab structures of concrete floors [20], and housing insulation [21]. Nowadays, the LCC has been quickly promoted as civil engineering construction materials with high flowability, low cement content, and high thermal insulation [13,22].

Lightweight cellular concrete has been commonly used in construction applications in different countries such as Germany, USA, Brazil, UK, and Canada [23]. Though there are limited studies regarding the practical applications of LCC in civil engineering, several road construction projects have been done with LCC due to its benefits as mentioned above. For example, LCC has been used as a subbase material in an industrial zone in the UK to replace the original layer, which consists of peat. Illinois also applies LCC in the road construction to provide a solution to the soft organic underlying soil, and it benefits the contractor by lowering unit cost, reduced construction time, and higher quality of material [19]. Applications of LCC are also found in Canada, and it was used as a subbase material of bus-lane rural road and highway [24].

LCC’s properties for application in civil engineering have been deeply studied. A critical task in LCC production is to control the nature, size, and distribution of pores, because the pore characteristic is the key factor to determine the density and strength of LCC [7,21,23,25]. Over the past few years, many studies have been done to improve LCC’s nature and its usage in structural applications [9,26,27,28,29,30]. These studies focus on the relationship between the microstructure and mechanical properties of LCC. Most studies analyzed the relationship of microstructure with compressive strength and modulus of elasticity, which are essential factors for the application of LCC in infrastructure construction. These pores of LCC are composed of interlayer pores/spaces, gel pores, capillary pores, and air voids, with pore sizes varying from nanoscale scale to millimeter scale [31]. Nguyen et al. [32] studied the influences of pore-structure and mortar properties on the behavior of geopolymer foamed concrete. Results showed that the pore-size has a profound effect on the material’s fracture resistance. Batool et al. [33] studied the distribution features of pore size in cement-based LCC. The results showed that the narrower the pore distribution is, the greater the conductivity and the smaller the density is. The investigation indicated that LCC strength decreases with the voids increase [34,35]. The prediction models on compressive strength were also investigated by some researchers. These findings are mainly based on the artificial neural network [36], extreme learning machine, and regression analysis based empirical models [37]. Kearsley and Wainwright [28] examined the relationship between porosity and compressive strength. They presented the mathematical models to reflect the influence of porosity on the compressive strength of the material. Wee et al. [38] proposed a spacing factor parameter to characterize the air-void system in concrete. It can be directly related to the average air void size. Results showed that the decrease of spacing factor and increase of the average air-void size resulted in the reduction of the compressive strength. Besides, Nambiar and Ramamurthy [5], along with Hilal et al. [1], examined the pore structure inside LCC and demonstrated that porosity was not sufficient to regulate the characteristics of LCC. Other pore characteristics—such as pore size, size distribution, shape, and cell thickness—should also be considered for a more detailed understanding of LCC material.

Though LCC has been applied in road construction, there are still lacking a complete and thorough guide of LCC. Mechanical properties of LCC at a specific low density (400 to 600 kg/m^3^) need to be adequately examined. The relationship between the microstructure and the mechanical properties of LCC also needs further analysis.

In order to obtain the microstructure of LCC, electron microscopes (EM)—such as secondary electron (SE) and X-ray computed tomography (CT X-ray)—were widely employed [1,39,40,41,42,43]. SE can be used to capture images with surface details, and CT X-ray can be used to view the three-dimensional external shape of specimens. The biggest advantage of EM is that they have higher resolution and higher magnification (up to 2 million times). While they have a range of disadvantages as well: they are costly; sample preparation is often much more elaborate; the space requirements are high, and operators require more training and experience. All these negatives mentioned above limit their flexibility in use. Compared with EM, although optical microscope (OM) has a significantly lower resolution, it is cheap to purchase, easy to operate small to carry. Some researchers have used a camera connected to an optical microscope to capture images of foamed concrete mixes and identified air-voids with diameters over 20 μm [1,40]. It means that OM can be used to capture the microstructure of LCC.

In this study, the correlation between pore characteristics and mechanical properties of LCC was investigated using both image processing technology and experimental approaches. The effects of local pore characteristics on the physical properties were examined from LCC samples with different densities at a microstructure level. For that purpose, a set of LCC samples with low densities were produced by using the Provoton foaming agent. In general, pores in LCC are secured by using either the pre-foaming or mix foaming methods [1,4]; the LCC specimens used in this study were generated using the pre-foaming method.

The pore characteristics, including pore distributions of LCC samples, were described using environmental scanning electron microscope (ESEM), which was used to capture clear images. Machine learning was used to identify pores, and then watershed segmentation method [44] was used to segment and identify the pores with irregular shapes. Finally, pore characteristics (area, size, and shape) were obtained. In order to capture the pore structure conveniently and fast, an industrial HD camera system (IHDCS) was developed, which was used to replace ESEM to obtain image samples of pore-structure in this study.

Mechanical properties of LCC with different densities—including compressive strength, modulus of elasticity, flexural strength, and splitting tensile strength—were tested. Then, the relationship between mechanical properties and pore characteristics (i.e., pore area, size distribution, and shape) was investigated.

## 2. Preparation of LCC Specimens and Mechanical Test

### 2.1. LCC Specimens

LCC materials with different densities are prepared to investigate the effect of the pore characteristics on the mechanical properties. CEMATRIX Inc. Canada (Calgary, Alberta, Canada) provided all test LCC specimens, which were with different densities. The densities of specimens used in this research are 400 kg/m^3^, 475 kg/m^3^, and 600 kg/m^3^, and each specimen is denoted as D400, D475, and D600, respectively. Foamed specimens are generated by the pre-foaming method, by which the pre-foamed foams are added into a base cement slurry mix until it achieved target density. The base mixes were composed by mixing general use Portland cement, Grade 80 NewCem slag [45], and water. The composition is 80% cement to 20% slag by mass, and the water to binder ratio is 0.5. The density of the base mix is 1823 kg/m^3^.

**Figure 1:**
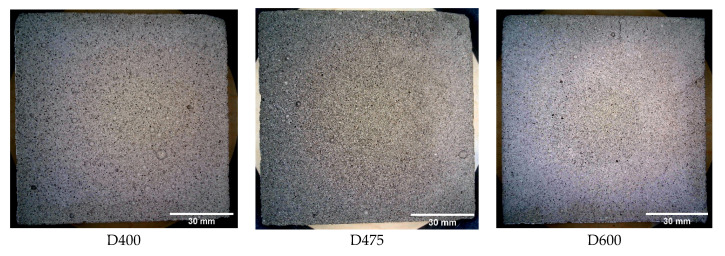
Lightweight cellular concrete specimens with different densities.

Figure 1 shows the LCC samples with different densities. There are two types of samples used in the study, cylindrical and beam samples. Here, these samples, which are used to measure the pore structure characteristics, are obtained by slicing beam samples. Firstly, an Industrial HD camera module connected to a computer was used to capture specimens’ images, and then the characteristics of pore were studied, such as number, area, perimeter, and shape descriptors (circularity, aspect ratio, roundness, and solidity). After that, all of specimens were cut into 10-mm cubes for scanning electron microscope imaging for high-resolution ESEM images with optimized sample size. Images captured by ESEM, which is a FEI Quanta 250 FEG at the Watlab of the University of Waterloo were also used to study the characteristics of pore. The results of ESEM images were compared with that of Industrial HD Camera images.

### 2.2. Mechanical Properties Test Method of LCC

LCC is a relatively new material, and currently, there are no test standards for it in Canada. Most of the laboratory tests were performed in accordance with standard procedures stated in ASTM or AASHTO standards. In the absence of standard testing protocols for LCC, test methods that had been devised for other materials, which are similar to LCC in various contexts, were adopted based on best practice or expert opinion. In this study, compressive stress, modulus of elasticity and Poisson’s ratio, flexural strength, and splitting tensile strength of LCC were tested in the University of Waterloo Center for Pavement and Transportation Technology (CPATT) lab using the Materials Testing System. Samples were cured for 28 days before testing in the humidity chamber of University of Waterloo.

Compressive strength was measured on four Φ 75 mm × 150 mm (Diameter × Height) cylindrical specimens stated in ASTM Standard C495/C495M- Standard Test Method for Compressive Strength of Lightweight Insulating Concrete [46] per each density.

Modulus of elasticity and Poisson’s ratio were measured in accordance with ASTM Standard C469/C469M—Standard Test Method for Static Modulus of Elasticity and Poisson’s Ratio of Concrete in Compression [47]. The standard sample size is Φ 150 mm × 300 mm. A total of three specimens were tested per each density.

Flexural strength was tested using simple beams with third-point loading, and the use standard was ASTM C78/C78M [48]. The samples measured 100 mm × 100 mm × 400 mm, with three specimens per each density.

Splitting tensile strength of LCC was also tested as the standard of ASTM C496/C496M [49]. Four samples per each density were used, and the dimension of cylindrical samples is Φ 150 mm × 300 mm.

**Figure 2:**
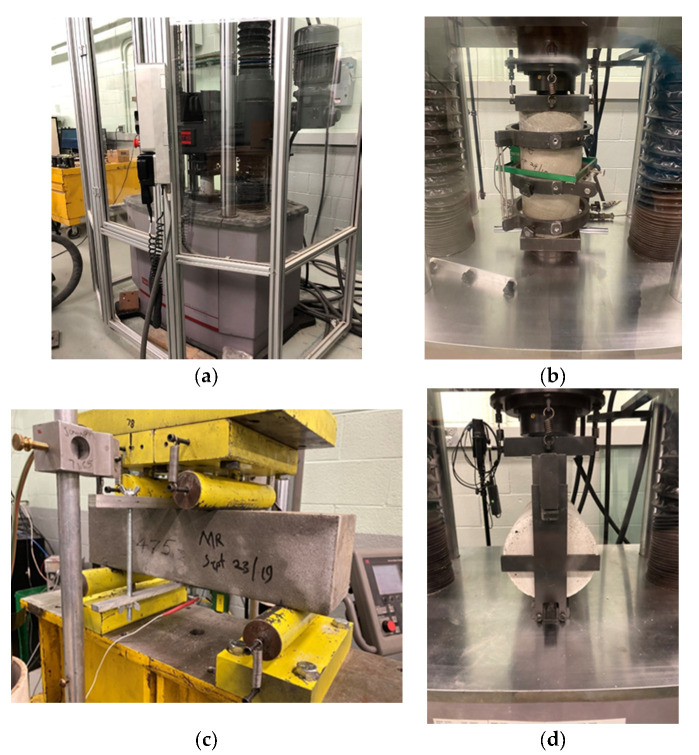
Experimental test set-ups of LCC. (**a**) Compressive strength test. (**b**) Modulus of elasticity test. (**c**) Modulus of rupture test. (**d**) Splitting tensile test.

Figure 2 shows the experimental test set-ups of LCC.

## 3. Image Capturing System and Image Processing Methodology

### 3.1. Industrial HD Camera System (IHDCS)

**Figure 3:**
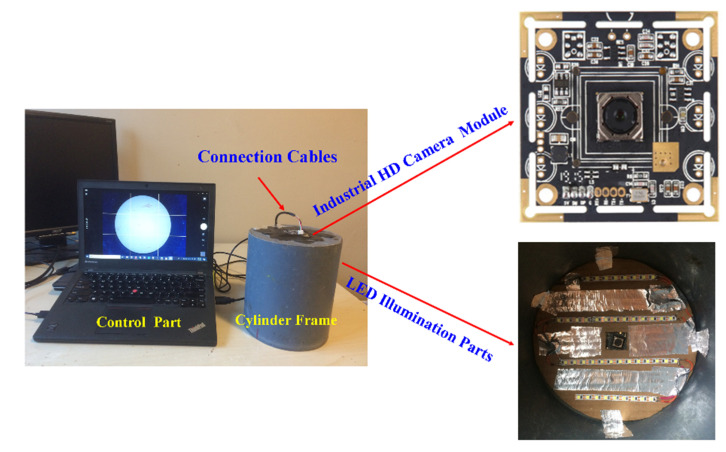
Industrial HD macro photography image capturing system.

**Figure 4:**
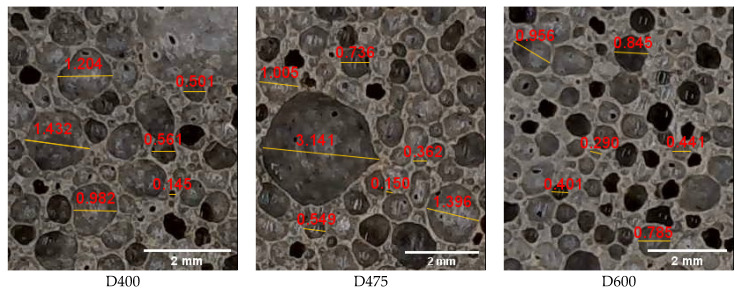
Images of specimens captured by IHDCS.

Scanning electron microscopy is appropriate for the pore structure and microstructure analysis of the foam concrete [50]. It can obtain clear photos of pores, and it is helpful to analyze the pore characteristics. Most ESEMs are able to scan image materials from 1 cm to 5 microns in width; however, sometimes, it is not big enough. Thanks to the development of technology, macro photography cameras are an attractive solution when compared to traditional large ESEMs typically found in university microscopy centers. In this study, an Industrial HD Camera System (IHDCS) was developed to capture the pore structure conveniently and fast. The system consists of capturing module (industrial HD macro photography camera module), illumination part (LED light), transmission part (connection cables) and control component (Laptop). The capturing module and illumination part are fixed on the top of a cylinder frame and connected with a laptop with cables. The HD macro photography camera module, ZJHY-179-R-01 AF, is produced by Ceyuan Inc. China, and its parameters are shown in Table 1. Figure 3 and Figure 4 separately show the capturing system and the captured images.

Figure 4 shows the pore images of samples, captured by IHDCS, with different densities. It can be observed that, relative to the high-density specimens (D600), the bubble size of low-density specimens (D400 and D475) have greater variability. Mostly, large-size bubbles are formed by merging adjacent small-size bubbles.

### 3.2. ESEM Imaging Method

**Figure 5:**
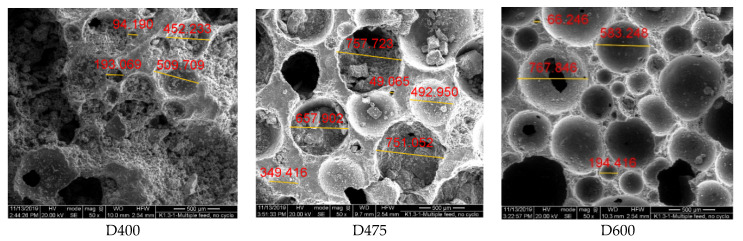
ESEM images with different densities.

In this study, for a more detailed examination of the pore characteristics of samples, environment scanning electron microscope (ESEM) images are utilized to examine the pore sizes and distributions of the LCC. The specimens are 1 cm cubes. Figure 5 shows the secondary electron images of the LCC specimens performed by ESEM. In these images, numerous spherical pores with different sizes in each specimen are identified. Compared to Figure 4, bubbles have sharper edges in the ESEM image. Similar to IHDCSs results, Pore sizes of specimens D400 and D475 are more variable compared with D600. Particularly, lots of bubbles in D400 connected to form irregular bubbles. Besides, the thickness between pores of D600 is obviously larger than that of D400 and D475.

### 3.3. Image Processing Methodology

**Figure 6:**
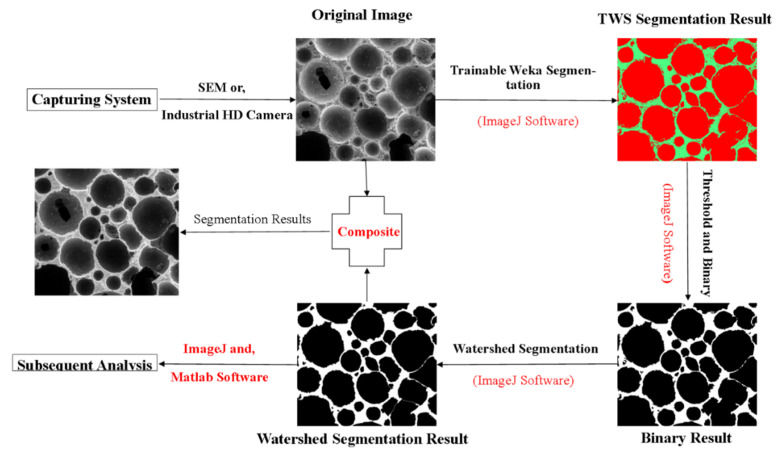
Image processing procedure.

FiJi ImageJ [51] software was used for the image processing of photographed LCC. FiJi is an image processing package, and it bundles a lot of plugins that facilitate scientific image analysis. In this study, the Trainable Weka Segmentation (TWS) plugin was used to segment the pores structure of LCC. TWS is an open-source project and combines a collection of machine learning algorithms with a set of selected image features to produce pixel-based segmentations. Firstly, the pores and solid area of the LCC sample were selected separately, which were used to training and got a suitable classifier. Once the classifier was obtained, it was used to classify the pore and solid area and got the TWS segmentation result, which is an 8-bit color image. Then, a threshold value was selected based on Otsu method [52] to transform the image into a binary image. In the binary image, black parts represent the voids or pores, and the white parts represent the solid part. In addition, watershed segmentation method was used to enhance the image segmentation quality. In this way, the contacting pores could be successfully segmented. The composite image of the original and watershed segmentation result was used to verify the effect of segmentation (more detailed information can be found in Figure 6). The watershed segmentation result was used to obtain the feature data of pore for the subsequent analysis.

## 4. Results and Analysis

### 4.1. ESEM Pore Characteristic Analysis

**Figure 7:**
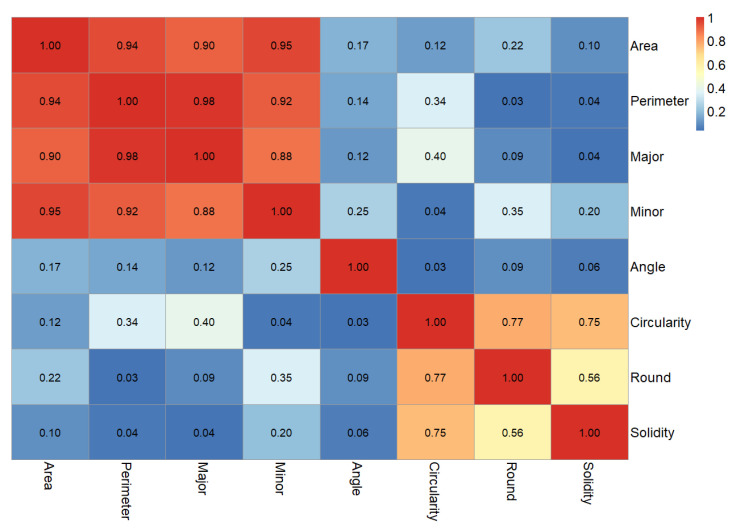
Heat map of correlation coefficient result.

Based on ImageJ software, parameters of the pore are easily obtained, such as area, perimeter, primary axis, and secondary axis of the best fitting ellipse and shape descriptors (circularity, roundness, and solidity). Firstly, the strength of the relationship between every two parameters was calculated, and the results are shown in Figure 7.

The heat map is helpful for showing variance across multiple variables, displaying whether any variables are similar to each other, and for detecting if any correlations exist in-between them. The number on each cell shows the correlation coefficient of each two parameters. The value range is from −1.0 to 1.0. If correlation equals to 1.0, it shows a perfect positive relationship. If correlation equals to −1.0, it also means a perfect negative correlation of two parameters. No matter perfect negative or positive correlation, they all show that parameters have a strong correlation. When correlation equals zero, it means that there is no relationship between the two parameters. In this study, correlation results were converted into non-negative values, which were used to consider the correlation among different parameters. Because the results of different specimens have a consistent trend, Figure 7 shows the result of D475 as an example. In Figure 7, a divergent color gradient defined by three hues (from blue to white to red) made the low and high ends of the range visually distinct. Increasing red hues represent stronger correlations between two parameters. In contrast, increasing blue hues mean weaker correlations. Obviously, parameters of pore area, perimeter, major and minor axis have a strong correlation between each other. Their lowest correlation coefficient value is 0.88. It means that the trends of pore area, perimeter, major, and minor axis have highly consistent. It is appropriate to adopt the pore area’s changing trend to represent those of the other three parameters. Although the shape descriptors (circularity, roundness, and solidity) have a high correlation between each other, the maximum of correlation is less than 0.8. Meaning that the changing trend of shape descriptors has certain differences. It is better to consider the shape descriptors’ changing trend separately. Therefore, the representative properties—area of pore, circularity, and solidity—were considered in subsequent analyses.

#### 4.1.1. Pore Size and Thickness of Solid Part

**Figure 8:**
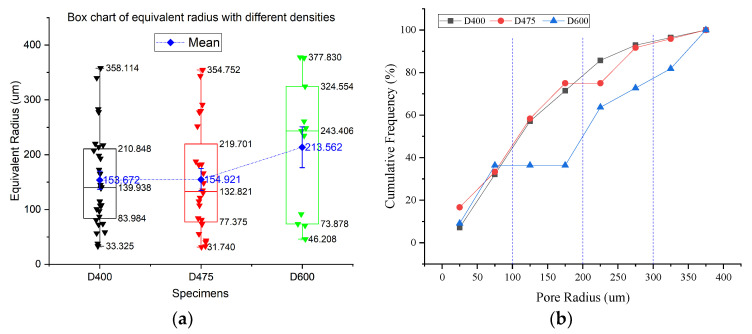
Box chart and cumulative frequency analysis of Equivalent radius. (**a**) Box chart of equivalent radius. (**b**) Cumulative frequency of pore radius.

Figure 8 shows the pore size of specimens with different densities. Figure 8a clearly shows that specimens of D400 and D475 have a similar distribution and which is different from that of D600. Firstly, the pore numbers of D400 and D475 are more than two times that of D600. The order of pore numbers is D400 > D475 > D600. Although there are more pores in specimen D400 and D475 than that of D600, most of them are small. Figure 8b presents that more than 70% of pores in D400 and D474 are less than 200 μm, while the percentage of D600 is only about 36%. On the other hand, there are also a considerable number of pores larger than 200 μm in D400 and D475. Besides, the number is similar to that in D600. Hence, the variance of pore size in D400 and D475 is higher than D600.

**Figure 9:**
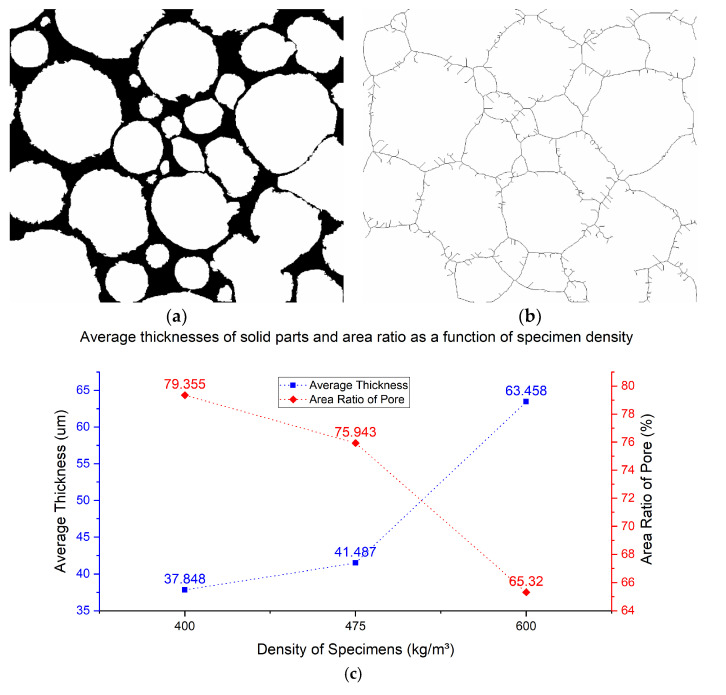
Average thicknesses of solid parts. (**a**) Inverted binary image. (**b**) Skeleton of the solid part. (**c**) Average thickness of solid parts and area ratio as a function of specimen density.

Figure 9 presents the average thickness results of the solid part in specimens. In order to obtain the average thickness of the solid part, firstly, the watershed segmentation result binary images of specimens were inverted, which were used to calculate the total area of solid parts. In the inverted binary images, the white areas are pores and the black area is the solid part, as shown in Figure 9a. Then, the inverted image was skeletonized, shown in Figure 9b. The Skeleton of the solid part can be obtained, which was considered to be approximately equal to the length of the solid part. Once the total area and length of the solid part were obtained, the average thickness was calculated, and the results are shown in Figure 9c.

As can be seen from Figure 9c, the average thickness has a positive correlation with density. It means that, when the density of the sample is low, the average thickness of the solid part is thin and vice versa. Relative to higher density samples (D600), the average thickness of the solid part in low-density samples (D400, D475) is reduced by about 40%. A small thickness value means a big pore area. The red curve in Figure 9c shows the trend of area ratio of pores with different densities of LCC. It is clear that area ratio decreases as density increases. Area ratio has a negative correlation with the density of sample.

#### 4.1.2. Shape Descriptors

**Figure 10:**
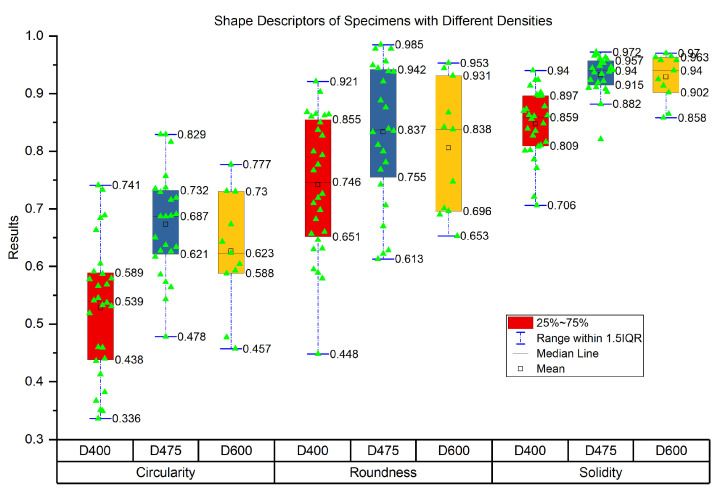
Data of shape descriptors of specimens with different densities.

Shape descriptors include circularity, roundness, and solidity. Circularity is used to describe how close the pore should be to a true circle, and it is defined as the ratio of the area of the object to the area of a circle with the same perimeter. Roundness is similar to circularity, and the difference is that roundness does not consider the local irregularities. Roundness is defined as the ratio of the area of an object to the area of a circle with the major axis. Solidity is used to measure the density of pore. It is defined as the ratio of the area of an object to the area of a convex hull of the object. Figure 10 shows results of shape descriptors with different densities.

Each dot in Figure 10 represents a pore. Comparing with the other two specimens, D475 has the best distribution of shape descriptors, which is followed by D600, and the worst is D400.

By comparing circularity results of three specimens, half the pores’ circularities of D475 are between 0.621 and 0.732, whereas that of D400 and D600 are separately from 0.438 to 0.589 and from 0.588 to 0.73. High circularity value means that pore is more close to a true circle. Hence, specimen D475 has a better foam structure, which is good for its mechanical properties.

The results of roundness show a similar trend with that of circularity. The difference is that roundness values are generally higher than values of circularity, because roundness ignores the effect of local irregularities of pore. Hence, circularity contains more shape information of pore, and in this study, circularity is used to evaluate the circle shape of the pore.

Solidity results of specimen D475 and D600 are obviously more significant than that of D400. As the pore becomes solid, the pore area and convex hull area approach each other, resulting in a solidity value of one. Thus, it means that pores in D475 and D600 are closer to the true circle than that of D400. In addition, larger solidity value also indicates that there are fewer touching pores in the specimen. This is consistent with the specimen’s photos shown in Figure 5. In this study, solidity is mainly used to evaluate the touching pores.

### 4.2. Image Processing Results Analysis of ESEM and IHDCS

**Figure 11:**
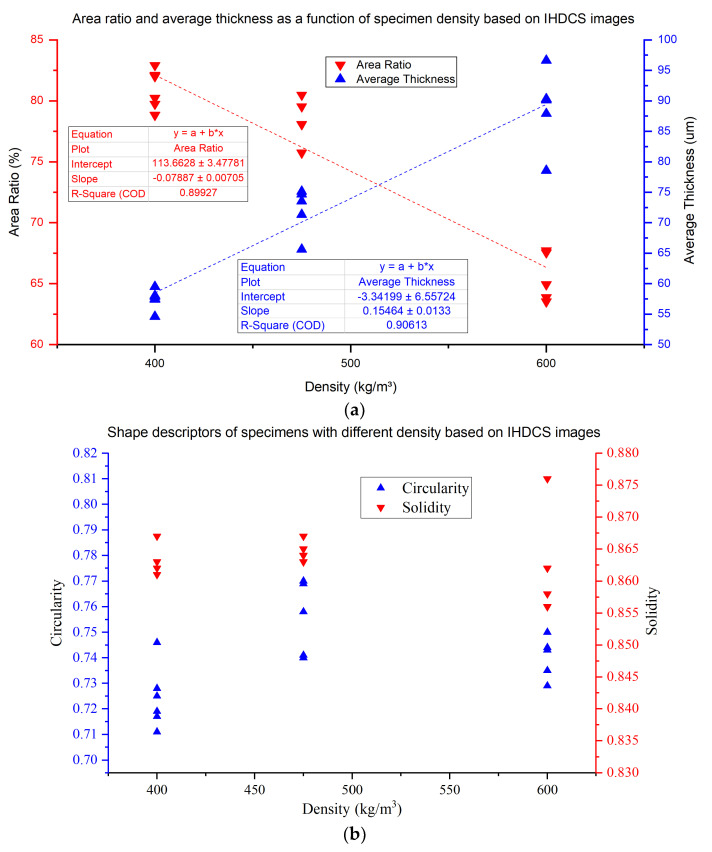
Results of pore characteristic based on IHDC. (**a**) Area ratio and average thickness as a function of specimen density based on IHDCS images. (**b**) Shape descriptors of specimens with different density based on IHDCS images.

There are six parallel specimens of each density, obtained by IHDCS, and test results of pore characteristics are shown in Figure 11. Figure 11a shows the results of area ratio and average thickness as functions of the density of LCC. Area ratio of pore and average thickness of solid parts are strongly correlated with the density of LCC. The increase of density is accompanied by the increase in the average thickness of the solid part and a decrease of the area ratio of pore. It can be observed that there are linear relationships for area ratio and average thickness, where area ratio is negatively correlated with density and average thickness is positively correlated with density.

**Figure 12:**
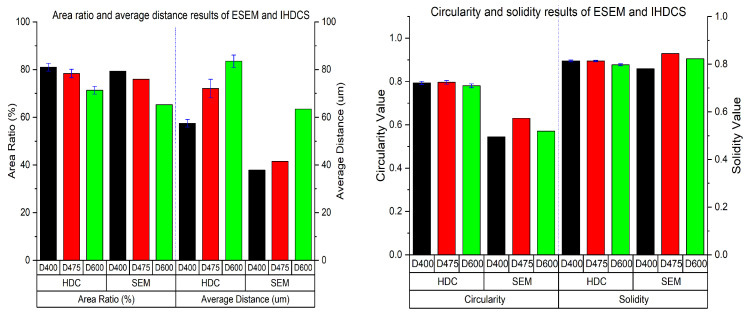
Comparative analysis of physical parameter results between ESEM and IHDCS.

Figure 11b shows the results of circularity and solidity. Specimen D475 has the best average results of circularity and solidity, which means that the shape of pores in D475 is more regular and closer to a true circle. Furthermore, compared to the other two specimens with different densities, D475 contains less number of touching pores. On the other hand, D400 has the worst results of circularity and solidity. It means that, due to the increase in bubble content, the inter-bubble spacing is reduced, and adjacent pores more easily contact each other. As a result, new irregular pores are generated, which causes the circularity and solidity to decrease. In order to verify the accuracy of the image processing results, results of IHDCS are compared with that of ESEM, and results are shown in Figure 12.

Figure 12 presents the comparison results of ESEM and IHDCS. In general, the results of IHDCS and ESEM have similar changes. In particular, the absolute value of pores’ area ratio and solidity results of IHDCS and ESEM are very close. Hence, IHDCS can be used to analyze the bubbles’ area ratio of LCC and the condition of touching bubbles, which can obtain a conclusion consistent with that of ESEM. On the other hand, results of the average distance and circularity of IHDCS and ESEM are different, although they show the same variation. Results of IHDCS are larger than that of ESEM, and the deviations of average distance and circularity are separately from 19.65 to 30.58 μm and from 0.126 to 0.179. The main reasons for the differences in results is that: on the one hand, relative to ESEM, IHDCS obtains lower resolution images, and the edges of identified bubbles are relatively smooth which lead to higher value of circularity; on the other hand, images captured by IHDCS have a larger range than that of ESEM, and each density considered six parallel samples. It means that IHDCS obtained more bubble samples than ESEM. It may be the cause of the difference in average distance results between IHDCS and ESEM. Furthermore, IHDCS results that considered more samples are more representative than that of ESEM.

### 4.3. Mechanical Analysis

**Figure 13:**
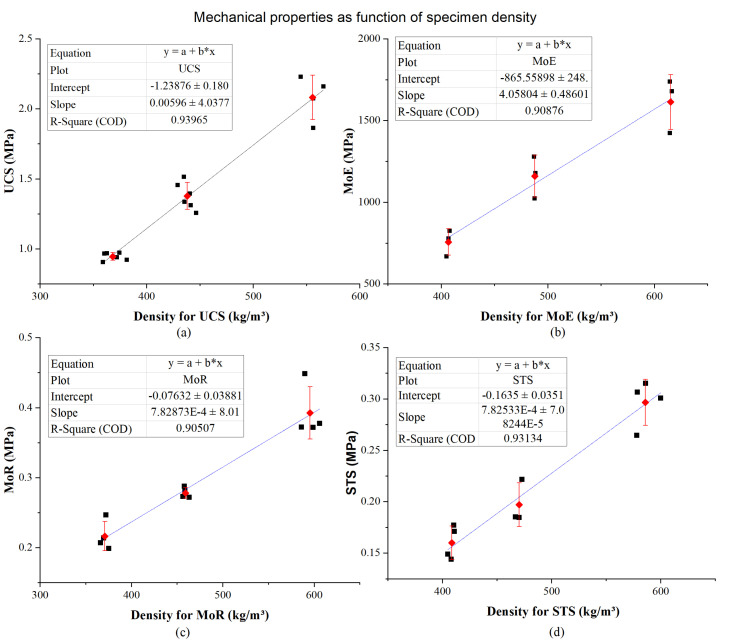
Mechanical properties as function of specimen density. (**a**) UCS as a function of specimen density. (**b**) MoE as a function of specimen density. (**c**) MoR as a function of specimen density. (**d**) STS as a function of specimen density.

The unconfined compressive strength (UCS), modulus of elasticity (MoE), modulus of rupture (MoR), and splitting tensile strength as a function of density are separately shown in Figure 13a–d. The increase in density is accompanied by an increase in mechanical properties, which shows a positive correlation between mechanical properties and density. The values of mechanical properties were approximated with linear functions, as shown in these figures.

The batch with a target density of 400 kg/m^3^ had the lowest mechanical properties, and it probably reflected the fact that there was weak foam structure when specimens with low density, on the other hand, specimens with higher density would have better or stronger foam structure.

Combining previous analysis of the pore characteristics of ESEM and IHDC image, three possible factors are affecting the mechanical properties of LCC. The area ratio of pore is an obvious factor. It is highly negative related to density, so there is a highly negative relationship between area ratio of pore and mechanical properties of LCC. When specimens with large area ratio of the pore, it means that samples have less solid part in the cross-section. It also means that the average thickness between pores is small. The decreased solid part leads to weak mechanical properties, because the solid part of LCC is the main part to bear the load.

Another factor is the shape of the pore. Results of circularity and solidity have shown that D475 and D600 have a better shape of pore than that of D400. Compared to D400, shapes of bubbles in D475 and D600 are closer to true circle and furthermore, both of them contain less touching bubbles. Regular bubble shapes and fewer touching bubbles are beneficial to the overall mechanical properties of the sample. Hence, the mechanical properties of D475 and D600 are better than D400.

Although the circularity and solidity of D475 are slightly better than those of D600, its mechanical properties are not better than those of D600. The main reasons are that: on the one hand, compared with D475, D600 has a larger solid thickness, which improved the mechanical properties; furthermore, the distribution of its particle size is more concentrated than D475, which is also better to its mechanical properties.

## 5. Conclusions

Based on the above image processing and experimental results, the following conclusions can be obtained.

(1) Based on the image processing results, IHDCS can obtain similar analysis results consistent with ESEM. Besides, compared with ESEM, IHDCS can capture a larger range of pore structures faster and more conveniently.
(2) There are strong correlations among pore area, perimeter, and major and minor axis. The lowest correlation coefficient value is 0.88; shape descriptors also have high correlation coefficients, but the maximum value is less than 0.8.
(3) Developed a method to calculate the average thickness of the bubble wall. The results show that specimens with high densities have a significant average thickness value. In this study, the average thickness value of D600 is more than 1.5 times that of low-density samples (D400, D475).
(4) The lower the density of the specimen is, the higher the ratio of the bubble area to the total test area. In this study, the pore area ratio of D400 is about 5% higher than that of D475. It is more than 20% higher than that of D600.
(5) The pore area ratio, average thickness of bubble wall, and shape directors (circularity and solidity) have strong correlations with the mechanical properties of LCC. These parameters could be useful to have estimations of mechanical behavior of LCC.

## Figures and Tables

**Table 1:** Parameters of Industrial HD camera module.

Name		Parameter and Description	Name	Parameter and Description
Module Size Focus		38 mm × 38 mm × 6 mm	Sensitivity	TBD
		AF	F/NO	2.5
Object Distance		5 cm-infinity	EFL	4.16 mm
Power Sensor Type		USB bus power	BFL	3.4 mm
		IMX179	FOV	80°
Active Array Size		3264 × 2448	TV distortion	<1.2%
Pixel Size		1.4 μm × 1.4 μm	IR filter	650 ± 10 nm
Maximum Image	15 fps	3264 × 2448, 2592 × 1944	Fixed pattern noise	<0.03%
	30 fps	1920 × 1080, 1280 × 720		
